# Exposure to bacterial and fungal bioaerosols in facilities processing biodegradable waste

**DOI:** 10.3389/fpubh.2022.789861

**Published:** 2022-11-16

**Authors:** Merja H. Kontro, Maija Kirsi, Sirpa K. Laitinen

**Affiliations:** ^1^Ecosystems and Environment Research Programme, University of Helsinki, Helsinki, Finland; ^2^Work Environment Laboratories, Finnish Institute of Occupational Health, Helsinki, Finland; ^3^Department of Occupational Safety, Finnish Institute of Occupational Health, Helsinki, Finland

**Keywords:** bioaerosols, bacteria, exposure assessment, fungi, microbial DNA, urban waste management

## Abstract

The aim of the study was to determine the exposure of workers within biodegradable waste processing facilities to bacteria and fungi to identify any exposures of potential concern to health. Occupational measurements were performed in six composting and three bioenergy (bioethanol or methane/biogas) producing facilities. Bioaerosols were measured from breathing zones with Button aerosol or open face cassette filter samplers, and swab specimens were taken from the nasal mucous membranes of the workers. *Aspergillus fumigatus, Bacillus cereus* group, *Campylobacter* spp., *Salmonella* spp., *Streptomyces* spp., and *Yersinia* spp. were determined by real-time polymerase chain reaction (qPCR). *A. fumigatus*, and mesophilic and thermophilic actinobacteria were also cultivated from filters. Bacterial airborne endotoxins collected by IOM samplers were analyzed using a *Limulus* assay. Bioaerosol levels were high, especially in composting compared to bioenergy producing facilities. Endotoxin concentrations in composting often exceeded the occupational exposure value of 90 EU/m^3^, which may be harmful to the health. In addition to endotoxins, the concentrations of *A. fumigatus* (up to 2.4 × 10^5^ copies/m^3^) and actinobacteria/*Streptomyces* spp. (up to 1.6 × 10^6^ copies/m^3^) in the air of composting facilities were often high. Microbial and endotoxin concentrations were typically highest in waste reception and pre-treatment, equal or decreased during processing and handling of treated waste, and lowest in wheel loader cabins and control rooms/outdoors. Still, the parameters measured in wheel loader cabins were often higher than in the control sites, which suggests that the use of preventive measures could be improved. *B. cereus* group, *Salmonella* spp., and *Yersinia* spp. were rarely detected in bioaerosols or nasal swabs. Although *Campylobacter* spp. DNA was rarely detected in air, as a new finding, *Campylobacter ureolyticus* DNA was frequently detected in the nasal mucous membranes of workers, based on partial 16S rDNA sequencing. Moreover, especially *A. fumigatus* and *C. ureolyticus* spp. DNA concentrations in swabs after the work shift were significantly higher than before the shift, which indicates their inhalation or growth during the work shift. Microbial qPCR analysis of bioaerosols and swab samples of nasal mucosa allowed measuring exposure in various work operations and during the work shift, identifying problems for health risk assessment to improve working conditions, and evaluating the effectiveness of preventive measures and personal protection of workers.

## Introduction

Microorganisms in biodegradable waste can cause infections, acute toxic effects, allergies, and cancer, which has been considered as a serious health problem in the increased recycling of waste material (1). The most common route of entry for biological hazards is inhalation as bioaerosols. Workers may be exposed to bioaerosols when microorganisms are released into the air during the handling of waste. Exposure to bioaerosols in waste management is associated with a wide range of health effects. According to interviews and follow-up studies, workers in the waste management sector experience more work-related symptoms and illnesses than other occupational groups (2, 3). Respiratory symptoms and lung impairment are the most widely studied and probably among the most significant bioaerosol-associated health effects. In particular, the aerobic treatment of waste in composting facilities is associated with adverse acute and chronic respiratory health effects for workers, such as pulmonary irritation and inflammation, chronic bronchitis, and occupational asthma (4–6).

The assessment of exposure to bioaerosols has traditionally been performed using culture-based methods. They are suitable for the simultaneous identification of many different species and are sensitive enough to detect spores of fungi and actinobacteria. However, cultivation is of limited utility in the quantitative assessment of exposure to many pathogens due to non-cultivability or even loss of viability, although cellular components may still induce adverse effects. In addition, pathogenic microbes can be hazardous at extremely low levels that cannot be detected by culture-based methods. Molecular-based diagnostic techniques have much better sensitivity than classical culture-based methods (7, 8). In the real-time polymerase chain reaction (qPCR) method, microbial deoxyribonucleic acid (DNA) as a cell component is a marker for the specific and rapid detection of microorganisms.

The nose is the primary entry route for inhaled air, and the first region of the respiratory tract to come into contact with bioaerosols. Therefore, qPCR detection combined with nasal samples from workers' mucous membranes could be used as a tool to assess exposure to airborne bacteria and fungi in waste management. PCR assays in studies of respiratory viruses and bacteria have provided similar results from both anterior nasal swab specimens and samples collected by nasopharyngeal aspiration (9, 10). Previous studies of nasal fungal and bacterial findings in occupational environments are scarce. Alsaleh et al. (11) have found mold in some nasal samples, when studying respiratory viruses in infants, despite using commercial swab tubes with antifungal agents. They speculated that the fungal findings were due to factors in the living environment of the infants. Furthermore, the carriage of livestock-associated methicillin-resistant *Staphylococcus aureus* to pig farmers and healthcare workers has been demonstrated (12, 13). Mbareche et al. (14) have shown correlation between the nasopharyngeal bacterial flora of swine workers and the biodiversity of swine barns bioaerosols. These studies demonstrate the transfer of microbes from the work environment to workers via inhalation.

Against the above background, the presence of endotoxins, and airborne microorganisms in composting and bioenergy facilities processing biodegradable waste, and then in the nasal mucous membranes of workers was investigated by cultivation and/or qPCR, with species identification by sequencing when necessary. *A. fumigatus* was measured as an indicator of workers' exposure to fungi, as it is a well-known cause of infectious mycoses, such as bronchopulmonary aspergillosis (15). Actinobacteria of the genus *Streptomyces* are Gram-positive bacteria, which produce resistant spores that can be infectious. Actinobacteria can promote the development of respiratory diseases together with fungi (16). *Bacillus cereus* group, *Campylobacter, Salmonella* and *Yersinia* species can be pathogenic bacteria especially in animal waste (17–20). The hypothesis of this study was that waste management workers become exposed to fungal and bacterial pathogens when inhaling bioaerosols through the nose, while the null hypothesis was that no exposure occurs when preventive measures are used. The relevance of the study was to characterize bacterial and fungal exposures for health care, which would lead to improved guidance in waste management companies, where the recycling of waste material is constantly increasing.

## Materials and methods

### Description of studied facilities

The occupational environments in which this study was conducted were six full-scale composting facilities, and three biogas or bioethanol producing facilities in Finland. Composting is an aerobic treatment in which organic biodegradable waste is recycled into soil fertilizer. Bioenergy facilities produce renewable energy from waste, after which the organic material can be further recycled into soil fertilizer or landscaping. Biogas, mainly a mixture of methane and carbon dioxide, is produced in waste management by anaerobic digestion, and bioethanol by waste fermentation. The facilities treated waste consisting of source-separated municipal biowaste, organic by-products from various agricultural and industrial (bakery, dairy, sugar production) processes, animal manure, and/or wastewater sludge. In the facilities, the transfer of biomass from one site to another was done with wheel loaders equipped with cabins to protect workers. The early stages of composting were indoors, followed by maturation in outdoor windrows (21). Sampling was performed in waste reception and pre-treatment, processing facilities, handling of treated waste, and then in areas with increased protection, including wheel loader cabins, control rooms, dining areas, and outdoors (field edges, parking sites).

### Sample collection

Bioaerosol samples from work environments were collected from different stages of the waste treatment processes at fixed measuring points approximately 1.5 m above the floor as close as possible to the process, from the workers' breathing zone, and from the wheel loader cabins. Reference samples were taken from presumed clean and safe work environments, such as a control room or outdoor air, at a distance of more than 300 m upwind from the composting area.

Bioaerosols were measured from workers breathing zones using a filter collection (22). Inhalable bioaerosols for cultivation (18–25 samples/microbe) and qPCR (44 samples) analyses were collected using a Button Aerosol Sampler (SKC Inc., Eighty-Four, PA, USA) at a calibrated flow rate of 4 liters/min in a sample pump with mixed cellulose ester filters (MCE, 1.2 μm, Ø 25 mm, SKC Inc.). The volumes of air samples collected ranged from 0.22 to 1.05 m^3^, the sampling time being the same as the duration of the work phase. In addition, inhalable bioaerosols were collected for cultivation (20–27 samples/microbe) and qPCR (65 samples) using open face cassettes with polycarbonate filters (PC, 0.4 μm, Ø 37 mm, Whatman International Ltd., Kent, UK) at a calibrated flow rate of 2 liters/min. The volumes of collected air samples varied between 0.11–0.54 m^3^ depending on the duration of the work. PC and MCE filters used for microbial cultivations were processed within 24 h of sampling, while filters used for qPCR analyses were stored at −80°C until analysis.

Altogether 48 nasal mucous membrane samples from both nostrils of 19 workers (27 samples from composting facility workers and 21 samples from researchers collecting the samples) were taken with nylon flocked swabs (Copan Innovation, Brescia, Italy), first in the morning from the nose of 17 workers, and then after the work shift in the afternoon from the nose of 31 workers. Parallel samples were taken from both nostrils. The swabs were rotated inside the nostril against the internal anterior walls and then placed in the provided transport tube. The swabs were stored at −80°C until qPCR analysis of bacteria and fungi.

All procedures involving humans, including environmental sampling, were conducted in accordance with the ethical standards of the institutional or national research committee, and the 1964 Declaration of Helsinki and its subsequent amendments or comparable ethical standards. The Ethics Committee of Helsinki University Hospital approved the study protocol (HUS/41/13/03/00/2011). All participants provided written informed consent prior to their participation.

### Microbial cultivations

Viable bacteria and fungi were cultivated for 3–14 days at specified temperatures on the following culture media. Thermotolerant fungi were cultivated on Rose Bengal (Hagem) agar (PC filter, 19 samples; MCE filter, 19 samples; 40 ± 3 °C); thermophilic actinobacteria on half-strength nutrient agar (PC filter, 23 samples; MCE filter, 18 samples; 55 ± 3 °C); and mesophilic actinobacteria (*Streptomyces* spp.), and other bacteria on tryptone-yeast extract-glucose agar (PC filter, 26 samples; MCE filter, 25 samples; 25 ± 5 °C) (23–25). After the incubation period, colonies were counted, and *Aspergillus fumigatus* and *Streptomyces* spp. were identified by microscopic examination of morphology. Concentrations of viable microorganisms in bioaerosols were expressed in cfu/m^3^ (colony forming units per cubic meter of air).

### Measurement of endotoxins

All gram-negative bacteria in the waste contain components called endotoxin in their cell walls. In this study, four parallel endotoxin samples were collected from air at 55 different sampling points using an IOM Sampler (SKC Inc.) at a flow rate of 2 liters/min (26). Biologically active endotoxins in air samples were measured using a validated kinetic chromogenic *Limulus* amebocyte lysate (LAL) assay (Kinetic QCL, Lonza, Walkersville, MD, USA). The collected glass fiber filters (1.0 μm, Ø 25 mm, SKC) were extracted with 5 ml of non-pyrogenic water for 1 h, after which the extracts were centrifuged at 1,000 x *g* for 15 min. Supernatants were diluted 1:25 (vol/vol) and analyzed in duplicate for the presence of endotoxin. Standard curves were performed by reconstituting the endotoxin standard of *Escherichia coli* O55:B5 in non-pyrogenic water. The spiked samples were analyzed to determine if inhibition or enhancement affected the endotoxin assay. Results were expressed in EU/m^3^ (endotoxin units per cubic meter of air).

### Real-time PCR

Real-time polymerase chain reaction (qPCR) was used to detect the DNA from cultivable and non-cultivable cells of *A. fumigatus, Bacillus cereus* group, *Campylobacter* spp., *Streptomyces* spp., *Yersinia* spp., and *Salmonella* spp. using the same experimental schemes as described in Rainisalo et al. (21). Bacterial and fungal DNA was isolated using the PowerSoil^®^ DNA Isolation Kit according to the manufacturer's instructions (Mo Bio Laboratories Inc., Carlsbad, CA, USA). A swab or filter was transferred to the PowerBead tubes instead of soil. Tubes were incubated at 70°C for 10 min to inactivate DNAses, followed by cell lysis for 3.5 min at 50 Hz using a TissueLyser LT bead mill (Qiagen, Valecia, CA, USA).

To monitor *A. fumigatus*, a 136 bp fragment of rDNA internal transcribed spacer 1 region was detected using primers AfumiF1 (5′-GCCCGCCGTTTCGAC-3′), AfumiR1 (5′-GTTGTTGAAAGTTTTAACTGATTAC-3′) and probe AfumiP1 (5′-CCCGCCGAAGACCCCAACATG-3′) (27). *B. cereus* group was followed using primers Pf 5′-GAGTTAGAGAACGGTATTTATGCTGC-3′ and Pr 5′-CTACTGCCGCTCCATGAATCC-3′, which detect 409 bp fragment of the cereolysin A gene (17). *Campylobacter* spp. (primers C412F, 5′-GGATGACACTTTTCGGAGC-3′; and C1228R, 5′-CATTGTAGCACGTGTGTC-3′; fragment size 816 bp) (18), *Streptomyces* spp. (primers StrepB, 5′-ACAAGCCCTGGAAACGGGGT-3′; StrepE, 5′-CACCAGGAATTCCGATCT-3′; fragment size 519 bp) (21, 28), and *Yersinia* spp. (primers Y.16S-86f, 5′-GCGGCAGCGGGAAGTAGTTTA-3′; B.16S-794r, 5′-TACAGCGTGGACTACCAGGGT-3′; fragment size 749 bp) (20) were monitored using PCR primers that detect the 16S ribosomal ribonucleic acid (rRNA) gene (16S rDNA). To detect the *Salmonella* species, primers 139 (5′-GTGAAATTATCGCCACGTTCGGGCAA-3′) and 141 (5′-TCATCGCACCGTCAAAGGAACC-3′) were used to amplify a 284 bp fragment from the invasion A gene (19). The detection limits based on the lowest positive results were ≤ 7 copies/PCR, corresponding to 101–1,853 copies/swab, and 890–4,069 copies/m3 depending on the filtered air volume. Internal amplification controls (IAC) were from kits designed for the microbes studied and purchased from Okepem Ltd (Finland).

The 20 μl PCR reaction mixture contained each primer: 0.5 μM (0.1 μM probe; *A. fumigatus*)/0.1 μM (*B. cereus* group)/0.4 μM (*Campylobacter* spp.)/0.4 μM (*Streptomyces* spp.)/0.23 μM (*Yersinia* spp.)/0.4 μM (*Salmonella* spp.); 1x DyNamo^TM^ HS SYBR Green qPCR Kit (contained hot start version of modified *Tbr* DNA polymerase, SYBR Green 1, optimized PCR buffer, 2.5 mM MgCl_2_, dNTP mixture) (Thermo Fisher Scientific Inc., Waltham, MA, USA); 2 μl of template DNA; and 1 μl of IAC. Real-time PCRs were performed using iQ5 Real Time PCR Detection System (Bio-Rad Laboratories, Hercules, CA, USA). At least four real-time PCR analyses were performed on at least two DNA isolations from filters and swabs. Controls in duplicate for each PCR experiment were as follows: reagents without template DNA and IAC (no amplification); reagents and IAC without template DNA (IAC amplified); and seven quantification standards in duplicate. The result was evaluated as negative when, according to melting curve analysis, the IAC was amplified without the product of the microbial primers.

To confirm the occurrence of *Campylobacter* spp. in the nasal samples, the amplified 16S rDNA sequences were sequenced as a purchased service at a DNA sequencing laboratory (Institute of Biotechnology, University of Helsinki, Finland) as presented in Pukkila et al. (29). The 16S rDNA sequences were compared to those in the gene library using the Fasta program. The 16S rDNA sequence accession numbers are OP432221-OP432236.

### Statistical analyses

Data were analyzed using SPSS Statistics for Windows, version 28.0.0.0 (IBM Corporation, Chicago, IL, United States). Endotoxin concentrations, and microbial DNA copy numbers were statistically analyzed using two-factor (operation, type of facility) Kruskal-Wallis test (KW) followed by pairwise comparisons using Mann-Whitney test (MW). Cultivated microbial numbers in composting facilities were analyzed by one-factor (operation) KW test followed by MW test. Differences in DNA copy numbers due to sampling methods; due to the high-efficiency particulate air (HEPA) filtration systems; before and after the work shifts; and in nasal swabs between facilities types were analyzed by MW. Differences in colony-forming units of *A. fumigatus* and actinobacteria due to sampling methods (Button aerosol or open face cassette filter samplers) were analyzed by *t*-test. Non-parametric statistical analyses were used, if the equality of variances (Levene's test) and/or normal distribution (Kolmogorov-Smirnov test) were not fulfilled. Pearson's two-tailed correlations for positive logarithmic values of colony-forming units and copy numbers were calculated.

## Results

### Endotoxins, and cultivated *A. fumigatus* and actinobacteria in bioaerosols

Endotoxin concentrations in bioaerosols were high, especially in composting operations compared to bioenergy (biogas or ethanol) producing facilities (KW/MW *p* ≤ 0.004; Table 1). Air endotoxin levels in the waste reception and pre-treatment were higher than during waste processing, and in wheel loader cabins and control sites (KW/MW *p* ≤ 0.034). The highest air endotoxin concentration of 16,000 EU/m^3^ was measured during the handling of the treated waste. However, the variation of air endotoxin concentrations was large during waste handling (<LOD−16,000 EU/m^3^), which is why this process stage did not differ significantly from the other operations. Air endotoxin concentrations in the wheel loader cabins ranged from below the detection limit to 33 EU/m^3^, with the exception of two poorly protected cabins with levels ranging from 1,600 to 8,400 EU/m^3^. Endotoxin concentrations in the air clearly exceeded the occupational exposure limit value of 90 EU/m^3^ in all studied composting facilities. In biogas facilities, this limit value was exceeded only once in indoor waste reception and pre-treatment.

**Table 1:** Ranges of variation for endotoxins; for plate counts of *A. fumigatus*, and mesophilic and thermophilic actinobacteria; and for copy numbers of *A. fumigatus, Streptomyces* spp., *B. cereus* group, *Campylobacter* spp., *Salmonella* spp., and *Yersinia* spp. DNA in composting and bioenergy production facilities.

**Operation**	**A. Waste reception and pre-treatment**	**B. Processing**	**C. Handling of treated waste**	**D. Wheel loader cabin**	**E. Control room, outdoors**
**Endotoxin (LOD 1–8 EU/m**3**)**					
1. Composting (EU/m3)	670–2,900 (5)^2, B, D, E^	3–390 (7)^2, A, E^	62–16,000 (4)^2^	< LOD−8,400(20)^2, A, E^	< LOD−4 (3)^2, A, B, D^
2. Bioenergy production (EU/m3)	8**–**370 (4)^1, B, D, E^	1**–**20 (3)^1, A, E^	< LOD (2)^1^	< LOD−8 (3)^1, A, E^	< LOD (4) ^1, A, B, D^
***Aspergillus fumigatus***					
1. Composting (cfu/m3)	4,914–20,915 (7)^D, E^	8,598–572,801 (9)^D, E^	311–23,804 (7)	172–8,785(13)^A, B^	1,195–1,783 (2)^A, B^
2. Composting (copies/m3)	5,099–62,767 (11)^3, B, C, D, E^	0–242,844 (13)^3, A, D, E^	0–7,137 (8)^3, A, D, E^	0–8,444 (8)^3, A, B, C, E^	0–120 (15)^3, A, B, C, D^
3. Bioenergy production (copies/m3)	0 (3)^2^	0 (8)^2^	0 (4)^2^	0 (4)^2^	0 (7)^2^
**Mesophilic actinobacteria**					
1. Composting (cfu/m3)	53,681–2,479,339 (7)^B, C, D^	129–63,174 (9)^A^	307–21,418 (8)^A^	123–17,193 (20)^A^	996 (1)
2. Bioenergy production (cfu/m3)	158–309 (2)	262 (1)	ND	ND	81–499 (3)
**Thermophilic actinobacteria**					
Composting (cfu/m3)	54–2,380 (7)^C, D, E^	4,435–118,256 (7)^C, D, E^	54–2,380 (7)^A, B^	156–3,361(15)^A, B^	548–3,782 (2)^A, B^
***Streptomyces*** **spp**.					
1. Composting (copies/m3)	51,526–1,645,675 (11)^2, B, C, D, E^	0–404,760 (15)^2, A, E^	0–70,885 (8)^2, A, E^	0–302,855 (27)^2, A, E^	0–3,981 (19)^2, A, B, C, D^
2. Bioenergy production (copies/m3)	0–5,289 (3)^1^	0 (8)^1^	0 (4)^1^	0 (4)^1^	0 (10)^1^
***Bacillus cereus***					
Composting (copies/m3)	0–430 (11)	0 (15)	0 (8)	0–1,054 (27)	0 (19)
Bioenergy production (copies/m3)	0 (3)	0 (8)	0 (4)	0 (4)	0 (10)
***Campylobacter*** **spp**.					
Composting (copies/m3)	0 (11)	0 (15)	0 (8)	0–802 (27)	0 (19)
Bioenergy production (copies/m3)	0–15,986 (3)	0 (8)	0 (4)	0 (4)	0 (10)
***Salmonella*** **spp**.					
Composting (copies/m3)	0–2,341 (11)	0 (15)	0 (8)	0 (27)	0 (19)
Bioenergy production (copies/m3)	0 (3)	0 (8)	0 (4)	0 (4)	0 (10)
***Yersinia*** **spp**.					
Composting (copies/m3)	0–2,068 (11)	0–2,739 (15)	0–8,624 (8)	0–11,781 (27)	0 (19)
Bioenergy production (copies/m3)	0–20,055 (3)	0 (8)	0 (4)	0 (4)	0 (10)

Samples were collected from waste reception and pre-treatment, processing, handling of treated waste, wheel loader cabins, and control rooms/outdoors; number of samples in parentheses. Superscript numbers indicate significant differences between composting and bioenergy production, and superscript letters show significant differences between operations (Kruskal-Wallis/Mann-Whitney, p ≤ 0.05).

ND, Not determined.

High numbers of *A. fumigatus* (up to 5.7 × 10^5^ cfu/m^3^) and actinobacteria (up to 2.5 × 10^6^ cfu/m^3^) were cultivated from composting facility air samples, regardless of sampling method (t-test, *p* ≥ 0.378; Button aerosol vs. open face cassettes; Table 1). *A. fumigatus* numbers in the air of composting facilities were highest in waste reception and pre-treatment and during processing, while significantly lower counts were cultivated from the air of wheel loader cabins, and control rooms/outdoors (MW, *p* ≤ 0.040). Similarly, thermophilic actinobacteria in the air of the composting facilities were most abundant in waste reception and pre-treatment, and also during processing, while significantly lower numbers were cultivated from the handling of treated waste, wheel loader cabins, and control rooms/outdoors (KW/MW, *p* ≤ 0.040). The concentrations of mesophilic actinobacteria in the air of the composting facilities were highest in waste reception and pre-treatment compared to lower quantities during waste processing and handling of treated waste, and in wheel loader cabins (KW/MW, *p* ≤ 0.040). In contrast, *A. fumgatus* could not be cultivated from the air of bioenergy facilities, and the numbers of mesophilic actinobacteria were low (81–499 cfu/m^3^).

### Microbial DNA in bioaerosols

The results of the qPCR assays correlated very well with the results obtained with culture-based methods for the identification and quantification of *A. fumigatus* (R = 0.696, *p* < 0.001, *n* = 25) and *Streptomyces* spp. (mesophilic actinobacteria *R* = 0.778, *p* < 0.001, *n* = 33; thermophilic actinobacteria *R* = 0.620, *p* < 0.001, *n* = 27) from air samples collected with a Button or open face cassette aerosol sampler. The air sampling method did not affect the DNA copy number results of any microbes (MW, *p* ≥ 0.084, Button aerosol vs. open face cassettes). *A. fumigatus* (up to 2.4 × 10^5^ copies/m^3^) and *Streptomyces* spp. (up to 1.6 × 10^6^ copies/m^3^) DNA levels were of the same order of magnitude as the concentrations of cultivable microbes in bioaerosols, and their levels in composting facilities were higher than in bioenergy production (KW/MW, *p* < 0.001; Table 1). No *A. fumigatus* gene copies were detected in the air of bioenergy producing facilities, and *Streptomyces* spp. DNA was only found at the waste reception and pre-treatment.

The number of *A. fumigatus* gene copies in composting facilities was highest in waste reception and pre-treatment, lower and about equal during processing and handling of treated waste, low in wheel loader cabin, and lowest in control room/outdoors (KW/MW, *p* ≤ 0.037; Table 1). In composting facilities, *A. fumigatus* DNA levels in well-sealed wheel loader cabins equipped with pressurization and high-efficiency particulate air (HEPA) filtration system were lower than in other wheel loaders equipped with poorly designed equipment (MW, *p* = 0.012). The highest *Streptomyces* spp. DNA copy levels in composting facilities were also observed in waste reception and pre-treatment, lower concentrations were detected during waste processing and handling of treated waste, and in wheel loader cabins, while numbers were lowest in control rooms/outdoors (KW/MW, *p* ≤ 0.004).

DNA from *B. cereus* group, *Campylobacter* spp., *Salmonella* spp., and *Yersinia* spp. was rarely detected in air samples from composting and bioenergy producing facilities (Table 1). *B. cereus* group and *Yersinia* spp. DNA was detected once, and *Salmonella* spp. DNA was detected twice in the waste reception and pre-treatment air of composting facilities. *Yersinia* spp. DNA was detected in the air once during compost processing, and twice during handling of treated compost waste. *B. cereus* group and *Yersinia* spp. DNA was detected in the air from the same wheel loader cabin where the highest endotoxin concentration was measured, while *Campylobacter* spp. DNA was found in two other wheel loader cabins at the composting facilities. The DNA of these bacteria was not detected in the control rooms or outdoor. In bioenergy facilities, *Campylobacter* spp. and *Yersinia* spp. DNA was detected only once in waste reception and pre-treatment.

### Microbial DNA in nasal samples

As the qPCR analysis of microbial DNA proved to be a reliable assay for air samples, the same methods were used to analyse nasal swabs. These samples were taken from the noses of 19 workers and sampling researchers before and after the work shift. In the composting facilities, *A. fumigatus* DNA was detected in the nasal swab samples of 9 workers after the work shift, and in only two samples before the work shift, the difference being statistically significant (MW, *p* = 0.042; Table 2). In the bioenergy producing facilities, nasal samples were taken from 10 workers, and only one worker produced a positive nasal finding for *A. fumigatus* DNA.

**Table 2:** The averages ± S.D., medians, and ranges of DNA copies of *A. fumigatus, Streptomyces* spp., *Campylobacter* spp., *Yersinia* spp., *B. cereus* group, and *Salmonella* spp. in nasal swab samples collected in composting facilities before and after the work shift, and in bioenergy facilities.

	**1. Composting (copies/m** ^**3**^ **)**		**2. Bioenergy** **(copies/m3)**
	**A. Before**	**B. After**	
***Aspergillus fumigatus***			
Average ± S.D.	13 ± 40^B^	118 ± 193^A^	8 ± 25
Median	0	0	0
Range	0–149	0–722	0–77
***Streptomyces*** **spp**.			
Average ± S.D.	394 ± 608^2^	1,569 ± 5013^2^	99 ± 169^1^
Median	66	319	0
Range	0–2147	0–23,887	0–465
***Campylobacter*** **spp**.			
Average ± S.D.	4,261 ± 6,834^B^	37,477 ± 937,784^A^	3,655 ± 5,780
Median	2,051	4,547	861
Range	0–21,406	0–415,745	0–18,433
***Yersinia*** **spp**.			
Average ± S.D.	73 ± 290	107 ± 502	Not detected
Median	0	0	
Range	0–1,160	0–2,356	
*Bacillus cerus*	Not detected	Not detected	Not detected
*Salmonella* spp.	Not detected	Not detected	Not detected

The superscript letters indicate statistically significant differences before and after shifts, and superscript numbers indicate significant differences between composting and bioenergy production facilities (Mann-Whitney, p ≤ 0.05).

More workers in composting facilities had positive *Streptomyces* spp. DNA findings than in bioenergy facilities (MW, *p* = 0.020; Table 2). A positive result was also observed in most composting plant workers at the beginning of the shift, but the mean amount of *Streptomyces* spp. DNA was about 4-fold higher after the work shift. In composting facilities, 8 positive results for *Streptomyces* spp. DNA were obtained from 16 nasal samples taken before the work shift, while the number of positive swab specimens after the shift was 18 out of 22. In bioenergy facilities, the proportion of positive *Streptomyces* spp. DNA swab samples was only three out of ten nasal samples.

The number of workers with a positive *Campylobacter* spp. DNA nasal sample was 17 of the 27 studied, although *Campylobacter* species were detected in only three air samples. Of these workers, 19 worked in composting facilities and 14 of them has a positive nasal sample. The quantities of *Campylobacter* spp. DNA in nasal samples after the work shift were significantly higher than before the shift (MW, *p* = 0.029; Table 2). Altogether 15 partial *Campylobacter* spp. 16S rDNA sequences were sequenced from nasal swab samples. All sequences were 99.9–100.0 % identical to the *Campylobacter ureolyticus* sequence. Partial 16S rDNA sequence of *Campylobacter* sp. DNA in an air sample from biogas production from pig manure was 99.9 % identical to the DNA of *Campylobacter lanienae* strain 15991b.

*Yersinia* spp. DNA was detected in the nose of only one worker, and no *B. cereus* group or *Salmonella* spp. DNA was found in nasal swab samples (Table 2).

## Discussion

### Microbial bioaerosols in air

Biodegradable waste provides a favorable substrate for microorganisms to live and reproduce. These microorganisms can be spread into the air by physically handling the waste, and a large portion of the bioaerosols can be present in the ambient air, as the results showed (Table 1). Bioaerosols formed from waste at composting facilities contained large amounts of mesophilic and thermophilic actinobacteria (*Streptomyces* spp.) and thermotolerant fungi (*A. fumigatus*), all of which play an important role in the transformation processes occurring during organic material decaying (30). Fungi of the genus *Aspergillus*, and actinobacteria have often been identified as dominant species at different stages of composting, often in the same quantities as measured in this study (7, 31–34). However, in this study, *A. fumigatus* was not detected in bioenergy facilities, although previously up to 1.2 × 10^4^ ITS genes/m^3^ have been detected mainly in waste reception and shredding, as well as in storage and output sites (35).

DNA of the other studied bacteria, *B. cereus* group, *Campylobacter* spp., *Salmonella* spp., and *Yersinia* spp., was detected in the air only rarely, more often in composting than in bioenergy facilities (Table 1). Nevertheless, *Bacillus* has been reported to belong to the dominant genus in compost bioaerosols (36, 37). In bioenergy facilities, *Yersinia* spp. DNA was detected only once in the air from the waste reception and pre-treatment simultaneously with *Campylobacter* spp. DNA. In composting facilities, *B. cereus* group, *Campylobacter* spp., *Salmonella* spp., and *Yersinia* spp. DNA was detected only in operations involving compost turning, either in the air of the composting space or in poorly protected wheel loader cabins. The concentrations of all studied microorganisms in the bioaerosols of the composting facilities were the highest in waste reception and pre-treatment, and among the lowest in the handling of treated waste, where concentrations could still be quite high. This is consistent with previous results, where bioaerosol concentrations have been highest during on-site agitation activities, such as turning, screening, and shredding (3).

In connection with microbial counts, it is important to recognize that only a small portion of microorganisms are cultivable or viable under bioaerosol sampling and culture conditions, resulting in lower numbers of colony-forming units than the actual total number of microbes. At the same time, large quantities of harmful biologically active cell components, such as endotoxins, can remain in the bioaerosols of composting operations (7, 8). Identification of common composting biomarkers and the causal relationships between bioaerosol exposure and disease outbreaks, as well as defining exposure limits are future requirements (8, 38). As large variations in microbial numbers were observed in this study, in agreement of other published results (3), it may be utmost important to recognize that the quality and treatment history of the waste material affects the microbial numbers and bioactive component quantities in bioaerosols. As unit operations in waste treatment can be difficult to standardize reliably, health risk assessment should be based on the highest commonly occurring concentrations in each operation.

In this study, bioaerosol levels were much higher in composting than in biogas or bioethanol producing facilities (Table 1). Dubuis et al. (39) have also shown that human exposure to bioaerosols in biomethanization facilities is lower than in composting plants. No legally binding limit values have been established for occupational exposure to bioaerosols (8, 38). However, data obtained from the assessment of occupational exposure to endotoxins, which serves as a marker for the whole group of Gram-negative bacteria, indicate that adverse health effects can be expected after chronic exposure to concentrations above 90 EU/m^3^ (40). The highest endotoxin concentrations (up to 16,000 EU/m^3^) were measured when compost was turned (Table 1), which is known to increase bioaerosol production (32). These results support previously published data suggesting that composting site workers may be exposed to high levels of bacterial endotoxins and microbial bioaerosols (31, 39, 41, 42).

Bioaerosols are a health risk, especially when waste is handled openly in the proximity of workers (32). Working inside a sealed cabin with air filtration had a major impact on reducing exposure to bioaerosols. Schlosser et al. (43) have found that, in particular, a pressurization and HEPA filtration system in the vehicle cabins of composting facilities can provide more than 99.9% protection efficiency. Their study emphasized that vehicle cabin protection systems reduced airborne endotoxins more than fungal spores. A similar observation was also found in this study that endotoxins were easier to remove than actinobacteria and fungi from the interior air of the cabins (Table 1). Regardless of the measurement methods, the concentrations of *A. fumigatus* and actinobacteria in the wheel loader cabins of the composting facilities were always significantly lower than in waste reception and pre-treatment. Still, the concentrations of endotoxins, as well as *A. fumigatus* and *Streptomyces* spp. DNA in wheel loader cabins were often higher than in control rooms or outdoors, suggesting that the hygiene could be better maintained.

### Microbial DNA in nasal samples

Composting plant workers had more *A. fumigatus* and *Campylobacter* spp. DNA in nasal samples after the work shift than before the shift. The difference in *Streptomyces* spp. DNA concentration in nasal samples before and after the work shift was less clear than the difference in *A. fumigatus* DNA. Most of the workers in composting plants had *Streptomyces* spp. DNA in the nose already at the beginning of the work shift. This means that workers may have been exposed to *Streptomyces* spp. elsewhere. Another possible explanation is that the concentrations of *Streptomyces* spp. DNA in the air (up to 2.5 x 10^6^ cfu/m^3^) and compost (21) in some waste handling operations were so huge that the nasal cells did not have enough time to disinfect the bacteria on the nasal mucous membranes between work shifts.

The most surprising result of this study was that *Campylobacter* spp. DNA was very common in the nasal mucous membranes of workers at composting facilities processing biodegradable waste. *Cambylobacter* spp. 16S rDNAs were sequenced to confirm their occurrence, and the most common species was *C. ureolyticus*. In Southern Ireland, molecular studies on the prevalence of various *Campylobacter* spp. in patients with gastroenteritis have identified *C. ureolyticus* as the second most common *Campylobacter* species (44). In other studies, this strain has been isolated from a wide variety of human samples, including superficial ulcers and soft tissue infections, urethritis, periodontal disease, and human feces (45, 46). *C. ureolyticus* has also been detected in the direct sequencing of 16S rDNA from template DNA isolated from human skin (47–49).

*Campylobacter* spp. DNA was detected in an air sample at a biogas plant processing pig manure. The 16S rDNA sequence of *Campylobacter* sp. in this air sample differed from those observed in the nasal swab samples. The bacterial DNA in the air sample was identical to *Campylobacter lanienae* DNA previously isolated from food-producing swine (50). Airborne cultivable *Campylobacter* spp. could not be recovered from bioaerosol samples in another agricultural study, although four of the 35 nasal flora samples from hog producers were *Campylobacter* spp. positive (51). The reason for this rare occurrence of *Campylobacter* spp. in air samples could be that, as preferentially anaerobic bacteria, they are easily destroyed in the air, especially during sampling. In this study, *Campylobacter* spp. DNA levels in air samples were low, close to the detection limit of 1.2 ± 0.6 x 10^3^ copies/m^3^ (1–5 copies/PCR). The workers may also have been exposed to *Campylobacter* spp. through skin contact with contaminated hands. Human campylobacteriosis is commonly considered to be a zoonotic disease with infection transfer being mainly mediated *via* the fecal-oral route (52). Exposure to bioaerosols may have promoted the nasopharyngeal growth of *C. ureolyticus* naturally present in the body, if not derived from bioaerosols.

Whether or not *Campylobacter* spp. are the leading cause of intestinal infections among workers in the waste management sector requires further studies. It is known that *Campylobacter* spp. are the major cause of foodborne diarrheal diseases in humans, and among the most common bacteria causing gastroenteritis worldwide (52). In both developed and developing countries, they cause more cases of diarrhea than *Salmonella* spp. (53). Therefore, *Campylobacter* species must be more common in the environment than is known. Our study, and new data from other studies suggest that *C. ureolyticus* is an emerging *Campylobacter* strain whose clinical importance is still underestimated (45).

DNA findings of other pathogens in nasal samples were sporadic compared to *Campylobacter* spp. DNA. *Yersinia* spp. DNA was detected in the nose of only one worker, who also had high numbers of *Yersinia* spp. DNA in the breathing zone in the cabin, collected by the air filter. In previous studies, *Yersinia enterocolitica* has been infrequently detected in both bioaerosols (2/18) and nasal secretions of hog producers (1/35) (51).

Detection of bioaerosols by the molecular qPCR assay was a sensitive method to assess workers exposure. Using nasal swabs in combination with the qPCR assay may be an even better choice for studying human exposure than taking air samples alone, as the ambient air is constantly inhaled through the nose. The air sample often represents only one part of the entire work shift. The importance of preferring the collection of nasal swab samples is supported by the observation that the difference in the microbial DNA of the nasal samples before and after the work shift was clear. In most cases, *A. fumigatus* DNA was not present in swab samples taken from the human nose before the work shift. In particular, in workers who did not work in waste management during the days before sampling, no *A. fumigatus* DNA was detected in the morning. Madsen et al. (54) have observed similar results; they measured the presence of fungi and bacteria in the noses of greenhouse workers using nasal lavage. They found that the concentration of fungi, β-glucan, and bacteria in the nasal lavage on Thursday at noon was higher than that on Monday morning. In addition, upper airway inflammation markers have increased in nasal lavage samples of composting facility workers after exposure to bioaerosols from Monday to Thursday (6). Thus, combining bioaerosol and nasal lavage microbial assays with the analysis of respiratory track inflammatory markers could improve human health risk assessment in waste treatment.

## Conclusions

The results showed that workers in biodegradable waste processing are exposed to bacteria and fungi that can be harmful to health. Especially in composting facilities, endotoxin concentrations in the air often exceeded the chronic exposure limit value of 90 EU/m^3^. Further, *A. fumigatus* and *Campylobacter* spp. DNA levels were significantly increased, and *Streptomyces* spp. DNA quantity was elevated in nasal swabs taken after the work shift compared to the levels before the shift. Workers' exposure to microbial hazards was high enough to lead to long-term respiratory diseases. Although the concentrations of endotoxins, *A. fumigatus*, and actinobacteria/*Streptomyces* spp. in wheel loader cabins were typically lower than in waste reception and pre-treatment, the levels were often elevated compared to the control rooms or outdoors, suggesting that hygiene could be better maintained. Waste management workers should be trained to take better care of hygiene, use appropriate protective equipment and all available preventive measures to preclude exposure to bioaerosol-related harmful microbial infections, such as campylobacteriosis.

Composting is well suited for the final treatment of organic process residues, although it has the problem of high bioaerosol concentrations. The number of harmful microbes was low in bioaerosols from closed bioenergy production processes. Therefore, composting could also be developed toward more closed processes to reduce worker exposure, while using wheel loader cabins with properly maintained HEPA filters and slight air overpressure. The studied facilities were simple in structure and the transfer of compost from one site to another was based on wheel loaders. Automation could reduce exposure to bioaerosols, although workers may face additional hygiene risks from process maintenance and repair interruptions.

The significance of qPCR assays is that they create new applications for determining exposure to pathogenic microorganisms, such as the new finding on the common occurrence of *Cambylobacter* spp. in waste treatment was made in this study. Sampling of workers' nasal mucous membranes shows the actual inhalation of microorganisms that may pose a potential risk to their health. These nasal samples can also be used to evaluate how effectively respiratory protection equipment protect workers from bioaerosols.

## Data availability statement

The original contributions presented in the study are included in the article, further inquiries can be directed to the corresponding author.

## Ethics statement

The studies involving human participants were reviewed and approved by all procedures that involved human participants including environmental sampling were conducted in accordance with the ethical standards of the institutional or national research committee and the 1964 Declaration of Helsinki and its later amendments or comparable ethical standards. The Ethics Committee of Helsinki University Hospital approved the Study Protocol (HUS/41/13/03/00/2011). All responders provided written informed consent prior to their participation. The patients/participants provided their written informed consent to participate in this study.

## Author contributions

SL and MHK designed research, sampling, and analysis methods. MK analyzed the samples and organized the database. SL wrote the first draft of the manuscript. All authors contributed to manuscript revision, read, and approved the submitted version.

## Funding

This study was financially supported by the Finnish Work Environment Fund (Project 110359) and by Environment Grant 2012 from the Finnish Ekokem Group.

## Conflict of interest

The authors declare that the research was conducted in the absence of any commercial or financial relationships that could be construed as a potential conflict of interest.

## Publisher's note

All claims expressed in this article are solely those of the authors and do not necessarily represent those of their affiliated organizations, or those of the publisher, the editors and the reviewers. Any product that may be evaluated in this article, or claim that may be made by its manufacturer, is not guaranteed or endorsed by the publisher.
